# orthoFind Facilitates the Discovery of Homologous and Orthologous Proteins

**DOI:** 10.1371/journal.pone.0143906

**Published:** 2015-12-01

**Authors:** Pablo Mier, Miguel A. Andrade-Navarro, Antonio J. Pérez-Pulido

**Affiliations:** 1 Centro Andaluz de Biología del Desarrollo (CABD), CSIC-UPO-JA. Facultad de Ciencias Experimentales (Área de Genética), Universidad Pablo de Olavide, ES-41013, Sevilla, Spain; 2 Faculty of Biology, Johannes Gutenberg University Mainz, Mainz, Germany; 3 Institute of Molecular Biology, Mainz, Germany; Aberystwyth University, UNITED KINGDOM

## Abstract

Finding homologous and orthologous protein sequences is often the first step in evolutionary studies, annotation projects, and experiments of functional complementation. Despite all currently available computational tools, there is a requirement for easy-to-use tools that provide functional information. Here, a new web application called orthoFind is presented, which allows a quick search for homologous and orthologous proteins given one or more query sequences, allowing a recurrent and exhaustive search against reference proteomes, and being able to include user databases. It addresses the protein multidomain problem, searching for homologs with the same domain architecture, and gives a simple functional analysis of the results to help in the annotation process. orthoFind is easy to use and has been proven to provide accurate results with different datasets. Availability: http://www.bioinfocabd.upo.es/orthofind/.

## Introduction

Finding homologous sequences allows the functional annotation of proteins by function transference, which is deduced because these sequences have a common evolutionary origin, and is often used as support for evolutionary studies [1–3]. Within homologs, orthologs are sequences that evolutionarily appeared from a speciation event, which provides a higher confidence in the process of function transference. In this way, function can be assigned to proteins of unknown function from their known orthologous proteins [4].

When searching homologs to a given sequence the first choice is usually the standard tool for homology search, BLAST [5]. But to collect all the sequences considered as homologs and also to discriminate the orthologs, manual processing based on score thresholds is necessary. In fact, the orthologs can be discriminated by executing a second BLAST against the genome/proteome from which the initial sequence comes from, and so completing an approach called Reciprocal Best Hits BLAST, RBHB [6].

The current growth of the sequence databases and the necessity for more automatized tools has been the basis of the emergence of a plethora of computational tools and databases of homologs and orthologs. These methods are mainly divided in two classes [7,8]: graph-based and phylogenetic methods. Graph-based methods perform pair-wise sequence comparisons between whole genomes using mainly BLAST, and then construct a graph with sequences as nodes and edges as similarity scores. Since they are based on BLAST, they only take in account sequence data, but fail in considering domain architecture. Phylogenetic methods analyze sequence trees to localize evolutionary events, such as duplications and horizontal gene transfers. Although they are more accurate for discriminating among different evolutionary relationships, they usually depend on human expertise and have a great computational cost. Moreover, despite important differences between these two classes of methods, they usually produce similar results [9].

In addition, there are databases that store groups of homologs and orthologs [10–12], but they are usually restricted to specific groups of organisms or data sources, or lack periodic updates.

A common problem of current approaches to search for homologs and orthologs is the lack of analysis about protein function, which is usually directly assigned without a review of its annotations [13]. This problem is increased when multidomain proteins are analyzed, whose domain specific functions may be incorrectly transferred to homologs lacking the corresponding domains [14].

Furthermore, some users simply want to find homologs or orthologs of a sequence of interest, to carry out a laboratory experiment with the reported sequences [15]. So, new easy-to-use approaches are necessary in order to allow the users to use their own sequences both as query and as database.

The lack of a universal and accessible tool for the automatic discovery of homologs, and specifically orthologs, including functional analysis, led us to develop a new computational tool. The application, which we have called orthoFind, is implemented in a public web application that takes a sequence and automatically searches in a reference database for homologs and orthologs, though it can start from a set of homologous sequences to facilitate the search. It also allows the user to search its own sequence datasets, reference proteomes, or EST databases. This allows searching for expressed sequences still not characterized as coding sequences. In addition, the multidomain problem is addressed by comparing the length of the candidate sequences and reviewing possible additional domains not corresponding to the homologous group. The results of orthoFind show the functions, pathways and domain architecture of the sequences found, which are divided in homologs and orthologs. The information provided in the results facilitates functional analyses and annotation of new or unannotated sequences. We illustrate the use of orthoFind with the analysis of the homologous proteins Smn and Spf30 involved in the splicing process, demonstrating how to use it to create new knowledge.

## Materials and Methods

### The orthoFind algorithm

To carry out an exhaustive search for homologous protein sequences, the orthoFind algorithm needs one or more query sequences, and mostly uses the exhaustive PSI-BLAST tool [5] to maximize sensitivity, and to discover the most ancient homologues.

**Fig 1:**
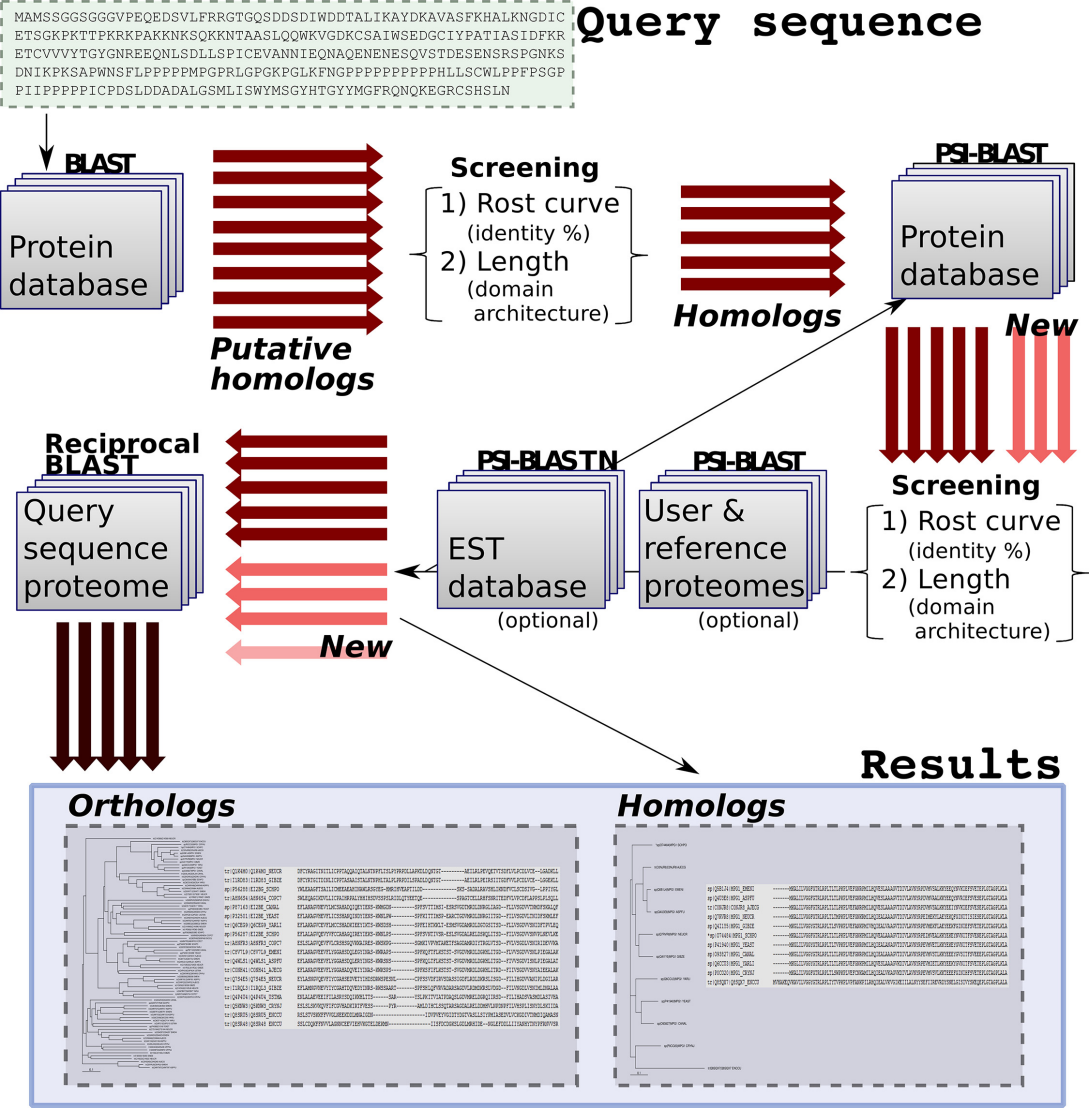
Steps followed by the orthoFind algorithm. It starts with a query sequence (or sequences) and a BLAST search to find putative homologs (represented by wide arrows). Then, the candidates have to pass through two filters: the Rost curve to remove sequences with low identity values, and the length filter to evaluate the domain architecture of the sequences. Next, a PSI-BLAST search can find new homologs, and the same filters are used again. Optionally, it is possible to look for additional homologs with a new PSI-BLAST search versus either a database of reference proteomes, sequences from the user or an EST database; these new results are also filtered by length and using the Rost curve. Finally, orthologs are discriminated by a RBHB, and the results are separated in homologs and orthologs, both of them displayed as a multiple alignment and as a phylogenetic tree.

It starts with an amino acid sequence and searches for its homologues in a protein database, using initially the BLAST tool (Fig 1). Alternatively, the user can provide a query composed of multiple homologous sequences. Thus, if these sequences have a wide evolutionary range, the search might find remote homologues.

Then, orthoFind uses both the user’s and found sequences as query for a PSI-BLAST search, to find homologous sequences in the selected database. The default database is Swiss-Prot [16] to encourage the use of curated sequences. Alternatively, the user can select a complete proteome from the UniProt database, a set of reference proteomes (see section 2.4 for details), or even its own database. If multiple sequences are provided as input, the first of the query sequences is considered as the reference sequence for the BLAST searches, and it will be the sequence used in the alignments to the database sequences, as well as the query sequence for reporting the identity percentages and expect values.

All putative homologs pass through two different filters before being considered as homologs.

- A putative homolog is only kept when the alignment with the reference sequence has a significant identity percent. To calculate the identity threshold, the Rost curve is used [17], which discriminates alignments based on both identity and alignment length. In the Rost equation, the identity threshold for an alignment of length L is defined as:
  $$p^{I}\;(L)=n+480\times L^{-0.32\times (1+e^{-L/100})},$$
  where *n* is a constant and represents a percentage of identity that is added to the curve to increase the stringency of the filter. For *n* = 0, the curve has an asymptote at identity of 20% around 100 amino acids in alignment length, but this value can be increased to 40% by using *n* = 20, which corresponds to a threshold for functional conservation [18].
- To consider a hit as a homolog it must have the same domain architecture than the true homologs. In this way, if a candidate lacks any domain, it will be discarded. As a proxy for recognizing similar domain architectures we use protein length, under the assumption that proteins with lengths differing more than the minimal length of a protein domain (*m*) will likely have different domain architecture. Thus, sequences with a length shorter or equal to the shortest homolog minus *m* will be removed by the filter. In addition, *m* is used to cut sequences with extra domains. So, if a candidate has a length higher than the longest homolog plus the *m* parameter, it passes through the filter but the extra domain (extra sequence of *m* amino acids in length) will be removed.

Both *n* and *m* parameters were optimized to get the best results (see next subsection).

To complete the set of homologous sequences, those passing through the filters are used in a new PSI-BLAST iteration, and this procedure is repeated until no more homologs are found.

Finally, candidates pass through a RBHB search in order to find the orthologous sequences, which are shown as a separate group in the results.

### Training and assessment of orthoFind

To get the most complete and specific set of homologs and orthologs, the cut-offs of orthoFind were optimized: the BLAST e-value, the minimal identity percent from BLAST alignment (*n*), and the minimum length for a domain region (*m*).

The training dataset consisted of 290 sequences randomly selected from the reference proteomes used by orthoFind, with length range from 50 to 1500 amino acids, in steps of 5 amino acids. This was done to obtain a training set unbiased in sequence similarity and length.

Finally, we illustrated the potential of the web application, using the Smn and Spf30 amino acid sequences from human (Q16637 and O75940) and *Schizosaccharomyces pombe* (Q09808 and O94519) extracted from the UniProt database. Multiple orthoFind analyses were performed, starting with different sets of queries and databases. To measure the accuracy of each test, the lists of gene names and annotations found were compared to those in the corresponding UniProt records. To compare the results of the different analyses and evaluate the matches, the Accession Numbers were used.

### Comparison of orthoFind against other methods

To compare the performance of orthoFind with other well-stablished approaches for searching orthologs, 100 different ortholog pairs were extracted from Ensembl database release 81 [19]. 25 pairs of orthologs from each of the following pairs of organisms were randomly selected: *Homo sapiens* (GRCh38.p3) and *Drosophila melanogaster* (BDGP6), *Mus musculus* (GRCm38.p4) and *Xenopus tropicalis* (JGI4.2), *Caenorhabditis elegans* (WBcel235) and *Rattus norvegicus* (Rnor_6.0), and *Danio rerio* (GRCz10) and *Bos taurus* (UMD3.1).

To run orthoFind, the amino acid sequence of the first organism in each pair was used as query sequence, and Animalia was selected as the search database. This database contains completely sequenced proteomes and includes unreviewed sequences from TrEMBL in addition to the reviewed sequences from Swiss-Prot.

For comparison, the search for orthologs was repeated with other published methods and databases, using default parameters: OrthoMCL v5 [20], EggNOG v4.1 [21], and OrthoDB v8 [10].

### Accuracy estimate

To measure the accuracy of orthoFind throughout this work, the homologs and orthologs found in a given orthoFind search are compared against the current knowledge from the curated Swiss-Prot or Ensembl databases. If a hit matches to any of the initial query sequences, the result is considered a true positive (TP), otherwise it is considered a false positive (FP). If the algorithm does not find the query sequence, this is considered a false negative (FN). These total number of TP, FP and FN are used to calculate the sensitivity (TP/(TP+FN)) and the specificity (TP/(TP+FP)) of a orthoFind search.

We also calculate the Relative Error Quotient (REQ), which evaluates the overall prediction quality by considering both the sensitivity and specificity measurements [22], as we have already used it in a functional annotation method [23]. In this case, REQ is calculated as:

$$REQ=\frac{FN+FP}{TP\times 2}$$

Low REQ values indicate a low error rate while high REQ values indicate a higher proportion of errors. This accuracy measure has the advantage of encompassing TP, FP and FN, thus combining sensitivity and specificity in a single value.

### Web application

To simplify the use of orthoFind, a web application was developed. It has been implemented using the Perl programming language and the CGI technology. The initial web form allows the upload of the query sequences and the selection of the database or databases. The orthoFind databases are Swiss-Prot (for curated sequences), and the reference proteomes (derived from genome sequencing projects) of 75 organisms from different taxa: *Archaea*, *Bacteria*, *Fungi*, *Plantae*, and *Animalia*. The user can also modify the sensitivity/specificity balance by changing the minimal identity percent threshold and the rest of the BLAST parameters, though the most accurate parameters are selected by default.

The results, separated in homologs and orthologs, include the list of candidates together with their Accession numbers, gene names, organism, length, identity to the reference sequence, and three kinds of functional annotations: pathways from the KEGG database [24], domains from the Pfam database [25], and GO terms [26]. Users may initially exclude GO terms that are not assigned by a curator, filtering by the IEA GO evidence code (Inferred from Electronic Annotation).

For each class of results the frequency of the different annotation terms is shown to allow the characterization of the sequence by the user. The percentages of pathways and domains are outlined to represent the putative biological process and domain architecture of the protein family. The GO terms are shown with a color range to highlight the most frequent annotations, which could be useful to annotate new sequences.

Finally, all protein sequences found are available for download in FASTA format, and a general view of the results is shown through a multiple sequence alignment and a phylogenetic tree, again separated in homologs and orthologs. The alignment and tree is created using the ClustalW algorithm, and the R library “ape” (Analyses of Phylogenetics and Evolution) is used to show the phylogenetic tree. These results are only available as a reference sample. The FASTA file with the sequences is provided so that the user can do further work, e.g. in both the alignment and the phylogeny.

## Results and Discussion

The orthoFind algorithm was initially optimized for maximizing its accuracy, and then it was assessed with a well-known set of proteins. Once it was validated, and the web application was created, orthoFind was tested through several analyses of pairs of homologous proteins presenting great sequence heterogeneity, proving the utility of this web application to solve biological problems.

### Optimization of orthoFind

To maximize the accuracy and the number of true homologs and orthologs in the final results, orthoFind selects the candidates by the expect value of BLAST alignments (e-value). It also discards non-homologous domains, and takes only alignments within an identity threshold by using an identity/length curve. Finally, it searches for orthologs by a RBHB strategy. All these steps were initially adjusted by optimizing the corresponding parameters.

**Fig 2:**
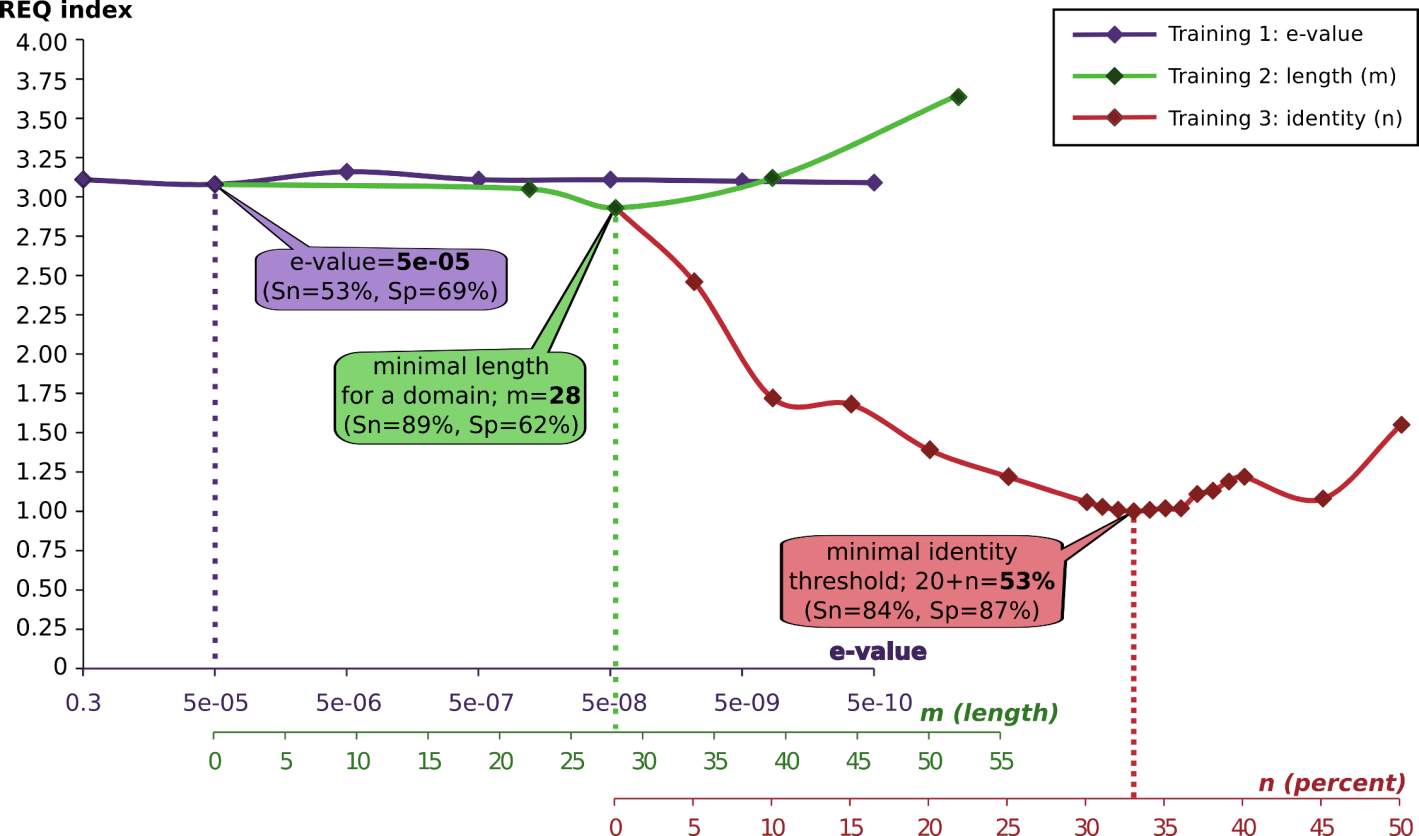
Accuracy measurement in the optimization of the orthoFind parameters. Different executions were carried out using a range of e-values (x-axis), m = 0, and n = 0. Then, the e-value was fixed to the value with the lowest *REQ* (dotted vertical lines), and new executions were carried out to fix the optimal length domain (*m*). Finally, the procedure was repeated with the identity (*n* in Rost curve). Values of sensitivity (Sn) and specificity (Sp) are shown on the balloons, which represent the points with lowest *REQ* and therefore with the best accuracy.

To perform the optimization and considering the heterogeneity in the potential query proteins, a sequence dataset with heterogeneous amino acid length (ranging from 50 to 1500 amino acids) was used. These sequences were randomly extracted from reference proteomes (see Methods for details). Thus, orthoFind was used to find homologs to these sequences exploring different ranges of values for each parameter. Finally, the accuracy was measured to find the values with best results considering the lowest REQ value, which reflects the best specificity and sensitivity properties, to maximize the accuracy (Fig 2).

When the best accuracy was achieved with a parameter, this specific value was fixed and the next parameter was optimized. Using this procedure, the optimized values were the following: 5e-05 as the e-value cutoff, 28 amino acids as the minimal length for a domain, and 53% as the minimal identity threshold.

From the training results, it is clear that the most important parameter is the identity of the alignments, which allows best accuracy when *n* is set to 33, and the minimal identity level gets to 53%. Even though the sensitivity has a slight drop from 89% to 84% after the optimization of *n*, the large increase in specificity allows a big improvement in accuracy.

Accordingly to these results, the values found in the optimization were set as default values for orthoFind. But considering the accuracy range allowed by *n*, we suggest that it can be changed by the user to fine tune the analysis and modify the sensitivity/specificity.

### orthoFind assessment and comparison with other methods

We assessed the accuracy of orthoFind for finding orthologous proteins in a practical test with sets of pairs of orthologs randomly extracted from the Ensembl database. One of the sequences of each pair was then used as query sequence with both orthoFind and other three well-established approaches for finding orthologs, OrthoMCL, EggNOG, and OrthoDB. The results obtained were similar with all the methods, with a minimum sensitivity of 76% when searching for *C*. *elegans* orthologs in *R*. *norvegicus* using OrthoMCL. However, not all species were available for searches in all methods; as a result OrthoMCL could not be evaluated with two of the species pairs, and orthoDB could not be evaluated with one of them (Table 1). The lowest specificity was obtained by EggNOG, which does not separate orthologous and homologous sequences in the results. Finally, most of the false positives found by orthoFind were due to redundant sequences found in the TrEMBL database (S1 File).

**Table 1:** Accuracy of orthoFinder and of another three methods measured with four sets of pairs of orthologs from different species. Results are shown for the different methods and sets, and the total accuracy is shown for each method. For each pair of orthologs, the sequence of the former species in the pair was used for searching the sequence of the latter one. When a method does not allow searching for orthologs in a specific species, a dash is shown. Sn = sensitivity (number of found orthologs / number of searched orthologs, and percentage), Sp = specificity (number of found orthologs / number of predictions, and percentage).

	orthoFind			OrthoMCL			EggNOG			OrthoDB		
Query / Subject	SN	SP	REQ	SN	SP	REQ	SN	SP	REQ	SN	SP	REQ
***H*.*sapiens / D*.*melanogaster***	25/25 (100%)	25/32 (78.1%)	0,140	25/25 (100%)	25/30 (83.3%)	0,100	24/25 (96%)	24/30 (80%)	0,146	23/25 (92%)	23/32 (71.9%)	0,239
***M*.*musculus / X*.*tropicalis***	22/25 (88%)	22/33 (66.7%)	0,318	-	-	-	25/25 (100%)	25/76 (32.9%)	1,02	-	-	-
***C*.*elegans / R*.*norvegicus***	21/25 (84%)	21/29 (72.4%)	0,286	19/25 (76%)	19/20 (95%)	0,184	24/25 (96%)	24/60 (40%)	0,771	22/25 (88%)	22/31 (71%)	0,273
***D*.*rerio / B*.*taurus***	23/25 (92%)	23/28 (82.1%)	0,152	-	-	-	25/25 (100%)	25/212 (11.8%)	3,78	25/25 (100%)	25/35 (71.4%)	0,2
**TOTAL**	**91/100 (91%)**	**91/122 (74.6%)**	**0,214**	**44/50 (88%)**	**44/50 (88%)**	**0,125**	**98/100 (98%)**	**98/378 (25.9%)**	**1,419**	**70/75 (93.3%)**	**70/98 (71.4%)**	**0,229**

**Fig 3:**
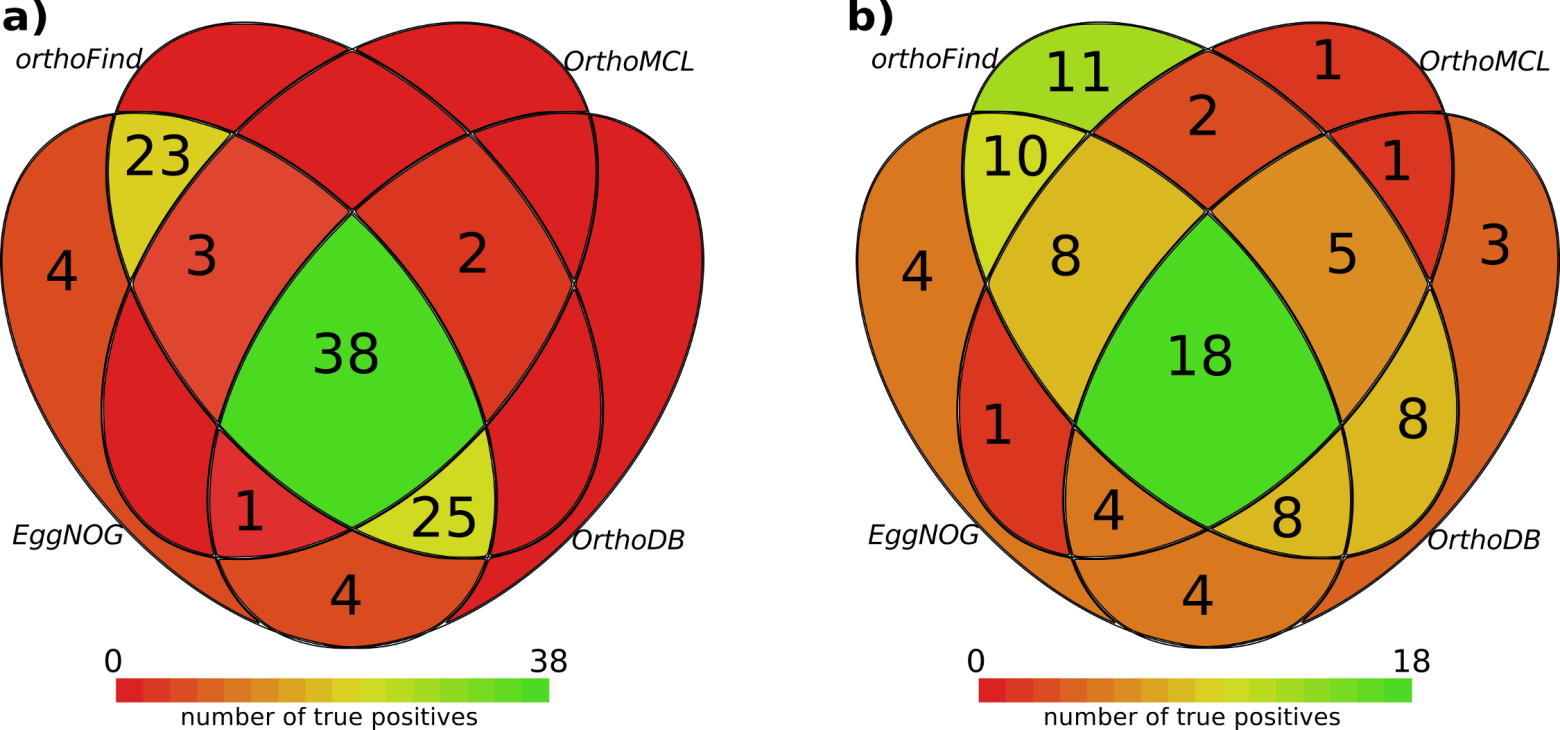
Overlap of the orthologs found between the different methods. a) Number of true positives found by the different methods, alone or jointly with some of the others. b) Number of true positives when unspecific results were removed (several orthologous candidates were predicted). The color scale highlights methods or groups of them with a high (green color) or low (red color) number of true positives. No number is shown if an individual method or group did not find any true positive.

The methods tested found most of the orthologs pairs with a good agreement: 38 out of the 50 pairs of orthologs tested were found by all methods (Fig 3A). However, some of the methods have low specificity; this is undesired if one seeks to discriminate true orthologs among possible false positives. Ideally, a method for the detection of orthology should find only one candidate. To test the methods from this point of view in a second comparison, we discarded results if a tested method found several orthologous candidates for a specific query sequence. According to this test, only 18 out of 50 possible orthologs were found by all the methods (Fig 3B), and orthoFind was the method with the highest sensitivity, as it found 11 orthologs that the other methods did not find, and it only missed 4 true positives that were found by the other methods.

In summary, though the best accuracy was obtained by OrthoMCL, it has a lower sensitivity than orthoFind which, together with EggNOG, were the only methods that allowed obtaining results from all the analysed species, though orthoFind was able to find orthologs in a more specific way. However, we note that while none of the methods was able to find the complete set of orthologs, collectively they only missed 12 orthologs in the high specificity test (Fig 3B). So, we recommend using several methods when the sensitivity is a key aim.

### Study of two paralogous proteins, Smn and Spf30, using the orthoFind web application

The Survival Motor Neuron protein (SMN, Q16637), related to the rare genetic human disease Spinal Muscular Atrophy, is involved in pre-mRNA splicing, and has also a specific but poorly understood function in motor neuron axons, including mRNA transport through microtubules and maturation of motor nerve terminals [27,28]. SMN orthologs have been found in all sequenced metazoans and also in filamentous and dimorphic fungi [29]. SMN has a paralog, known as SPF30 (O75940), which shares with SMN a Tudor domain that allows binding to splicing ribonucleoproteins [30], but SPF30 has not been involved in axonal function or development. This domain is also present in other proteins involved in the splicing process such as the Tudor domain-containing 3 protein (TDRD3, Q9H7E2) [31].

We have used orthoFind to find homologs and orthologs for both Smn and Spf30 in complete proteomes (reference proteomes in the web application), and to illustrate how to find new knowledge using its web application. Smn from *H*. *sapiens* and *S*. *pombe* were initially used as query sequences in two different searches. The global sequence identity between these orthologs is below 20%, which makes it difficult to find each other in an orthology search.

**Fig 4:**
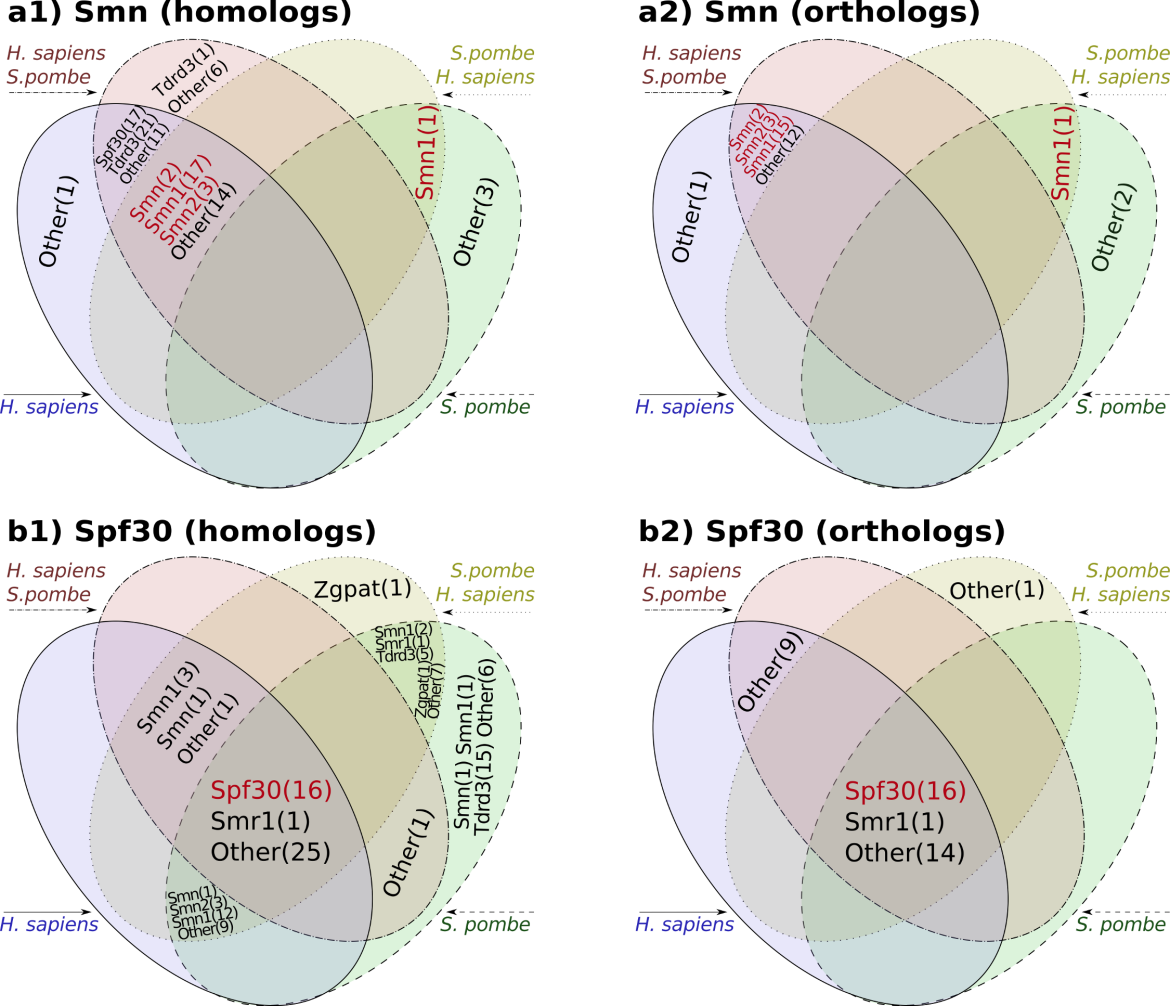
Gene names obtained when finding homologs and orthologs to Smn (a1 and a2, respectively) and Spf30 (b1 and b2, respectively) proteins using orthoFind and different query sequences. Four executions were made for each group using either one of the following query sequences: *H*. *sapiens*, *S*. *pombe*, *H*. *sapiens* plus *S*. *pombe* (using *H*. *sapiens* as reference for the alignments), or *S*. *pombe* plus *H*. *sapiens* (using *S*. *pombe* as reference for the alignments). Matching genes between the different executions are represented by the Venn diagrams, including the gene names and their number of occurrences (within parentheses). Gene names matching with the query name are highlighted in red color. Other = Uncharacterized proteins.

The search with human Smn found a high number of homologs with the gene name Smn, together with some Spf30 and Tdrd3 paralogs that were not present in the orthology prediction, as it was expected (Fig 4A, two bottom sets in every Venn diagram). Other uncharacterized proteins were also found, which have typical features of Smn, and therefore could be annotated as Smn. For example, early versions from *Pan troglodytes* UniProt:H2QR14 had the gene name ENSG00000172062, but in October 2014 the gene name was updated to Smn1. However, only four homologs and three orthologs were found using *S*. *pombe* Smn as query sequence, which represent the only fungal Smn proteins found, since the results from both tests did not overlap.

Two more executions were carried out to try to find together metazoan and fungal homologs and orthologs. To do that, both Smn proteins together were used as query, but once using *H*. *sapiens* Smn as reference, and another using *S*. *pombe* Smn. Thus, the majority of homologs previously found with *H*. *sapiens* Smn as query sequence were found again (Fig 4A, two top sets in every Venn diagram). But when *S*. *pombe* Smn was used as reference, only Smn proteins were found, but not the Spf30 and Tdrd3 paralogs.

The experiment was repeated with Spf30 proteins, of which *H*. *sapiens* and *S*. *pombe* orthologs share less than 20% sequence identity. In this case, all four searches for homologs agreed in finding Spf30 proteins (Fig 4B1), together again with uncharacterized proteins, and with an isoform from a *Caenorhabditis elegans* predicted protein named Smr-1 (Q7Z1Q5 and Q95Y51), with 31% identity to human Spf30. However, when only one query sequence was used, the results gave also false positive Smn and Tdrd3 proteins, and a transcription regulator from *Danio rerio*, named Zgpat, with a very similar N-terminus to Spf30, suggesting that the use of two queries can narrow the results and give more specificity. In addition, the specificity was still higher in the orthology results, where the gene name Spf30 was found in the majority of the cases, together with uncharacterized proteins with features from this protein (Fig 4B2).

**Fig 5:**
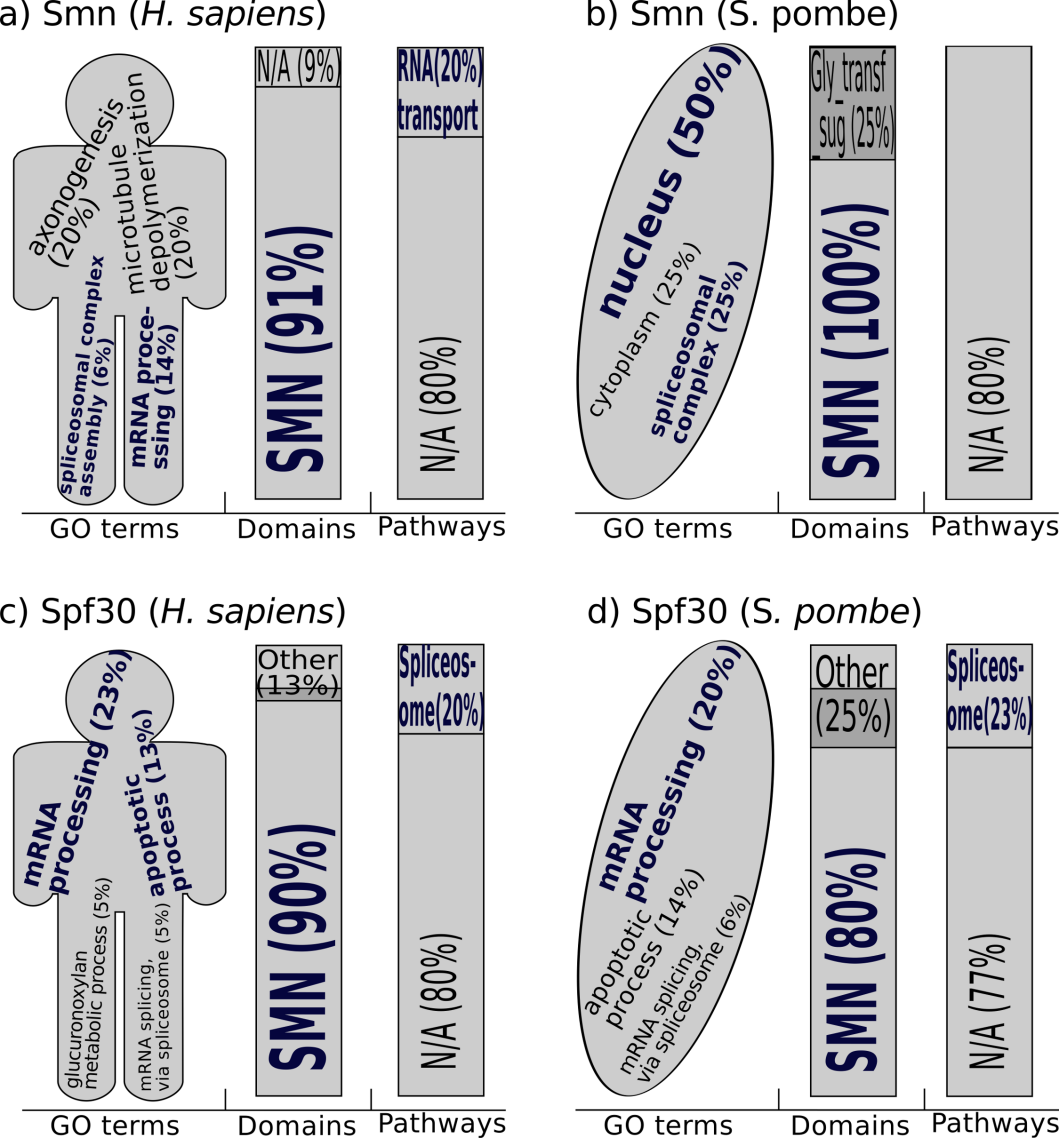
Annotations from the Smn and Spf30 orthologs found by orthoFind. The annotations were extracted from the results of the analyses described in Fig 4 when only one query sequence was used. Terms highlighted in blue color represent known annotations for these proteins. The relative frequency of each annotation reported by the web application in the results is shown within parentheses, and the letter size is correlated with its frequency. Only annotations with frequency higher than 5% have been taken in account, and only GO terms from biological processes have been used, except for *S*. *pombe* Smn, where cellular component was used because of the lack of other annotated GO terms. N/A = not available domain or pathway.

The web application of orthoFind also offers functional annotations that allow finding new knowledge. For example, when orthoFind was used with Smn and Spf30 (both from *H*. *sapiens* and *S*. *pombe*), the “SMN” domain was found in the majority of the orthologs, but the pathways were distributed between “RNA transport” for Smn, and “Spliceosome” for Spf30 (Fig 5), as it was expected by the known annotations of these proteins. Furthermore, the GO terms found match those known for these proteins such as “mRNA processing”, or “spliceosomal complex assembly” for Smn, or “apoptotic process” for Spf30. But other GO terms were found that are not present in the UniProt annotation of these proteins, such as “axonogenesis” and “microtubule depolymerization” for Smn, or “mRNA splicing, via spliceosome” for Spf30, despite of being biological processes related to the analyzed proteins [32,33], which would prove the accuracy of orthoFind.

## Conclusions

We have presented a quick and easy-to-use computational tool that can automatically search for homologous and orthologous protein sequences. It allows the user to obtain results within a few minutes, and without previous knowledge about sequence analysis. These results come together with annotation information, useful to gain knowledge about specific proteins, and a plain phylogenic study, useful, for example, to discard putative false positives. All of this allows using orthoFind for both evolutionary and functional studies at a genomic level without great effort.

## Supporting Information

S1 File Sequences found in the comparison of orthoFind, and another three methods for searching orthologs. (ODS)
